# Giant Brunner's gland hamartoma diagnosed via endoscopic mucosal resection: A case report

**DOI:** 10.1002/deo2.65

**Published:** 2021-10-24

**Authors:** Sakiko Naito, Masakatsu Fukuzawa, Shunsuke Nakamura, Shin Kono, Jun Matsubayashi, Takao Itoi

**Affiliations:** ^1^ Department of Gastroenterology and Hepatology Tokyo Medical University Tokyo Japan; ^2^ Department of Human Pathology Tokyo Medical University Tokyo Japan

**Keywords:** Brunner's gland hamartoma, duplication cyst, endoscopic mucosal resection, intestinal neoplasms, intussusception

## Abstract

We report the case of a patient with a giant Brunner's gland hamartoma that was pathologically diagnosed by endoscopic mucosal resection. A 69‐year‐old woman presented with intermittent abdominal pain, and imaging revealed a smooth saccular submucosal tumor, 40 mm in diameter, on the anterior wall of the duodenal bulb. Brunner's gland and smooth muscle tissue were observed on endoscopic ultrasound‐guided fine‐needle aspiration biopsy, which resulted in the preoperative diagnosis of a duplication cyst. However, subsequent endoscopic mucosal resection established a final histopathological diagnosis of Brunner's gland hamartoma.

## INTRODUCTION

Brunner's gland hamartoma (BGH) is a rare condition that accounts for approximately 0.01% of benign duodenal diseases.^1^ Endoscopic mucosal resection (EMR) of BGH should be considered if intussusception (causing abdominal pain and vomiting) or ileus is suspected or if anemia is present.^2^ Preoperative evaluations are performed using computed tomography (CT) or endoscopic ultrasound‐guided fine‐needle aspiration biopsy (EUS‐FNAB); however, it is difficult to make a definitive diagnosis.^2^ EMR, which is a minimally invasive diagnostic and therapeutic procedure, is more useful for establishing a final pathological diagnosis.^3^

## CASE REPORT

A 69‐year‐old woman presented with a history of occasional epigastric pain since her early childhood, but she had no symptoms, such as vomiting or anemia. Moreover, there was no particular history of illness. The patient was referred to the hospital because the screening endoscopy revealed a duodenal submucosal tumor. No abnormalities were observed in the physical examination and blood sample results. Upper gastrointestinal endoscopy revealed duodenal abnormalities, including a 25‐mm smooth saccular submucosal tumor on the anterior wall of the bulb. On endoscopy, we encountered a positive cushion sign and made a diagnosis of Brunner's gland hyperplasia. Therefore, the tumor was carefully monitored through annual endoscopy for changes in size and morphology.

**FIGURE 1:**
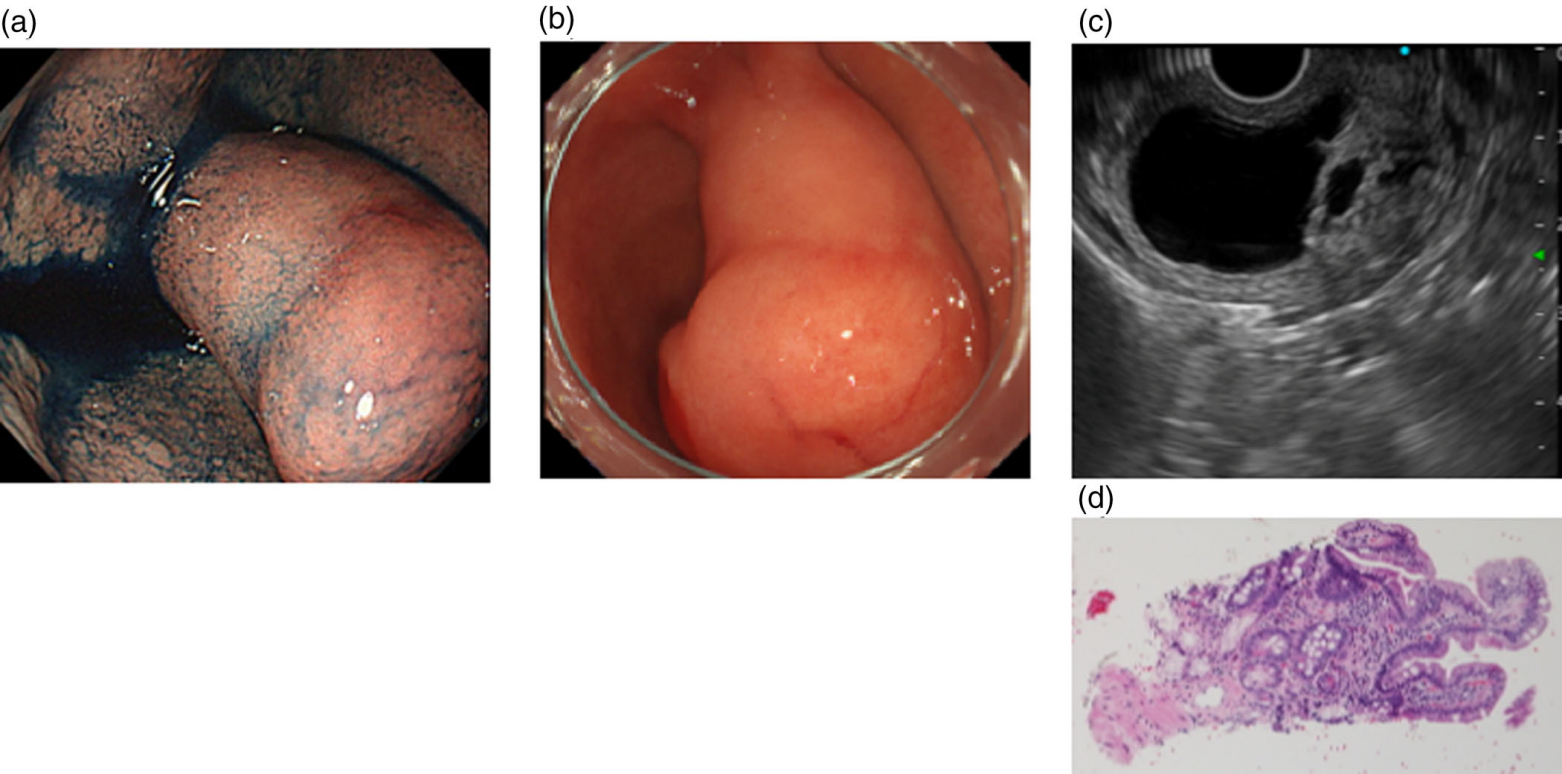
Endoscopic images and pathological findings of the lesion in the duodenal bulb: (a) A 25‐mm‐large duodenal submucosal tumor (SMT) was observed on the interior of the duodenum at the time of initial examination. (b) Follow‐up endoscopy findings after 2 years revealed an increase to 40 mm. (c) A hypoechoic lesion with non‐uniform hyperechoic components internally was detected by endoscopic ultrasound. (d) Histopathological examination revealed Brunner's glands and a smooth muscle tissue in the preoperative biopsy specimen

**FIGURE 2:**
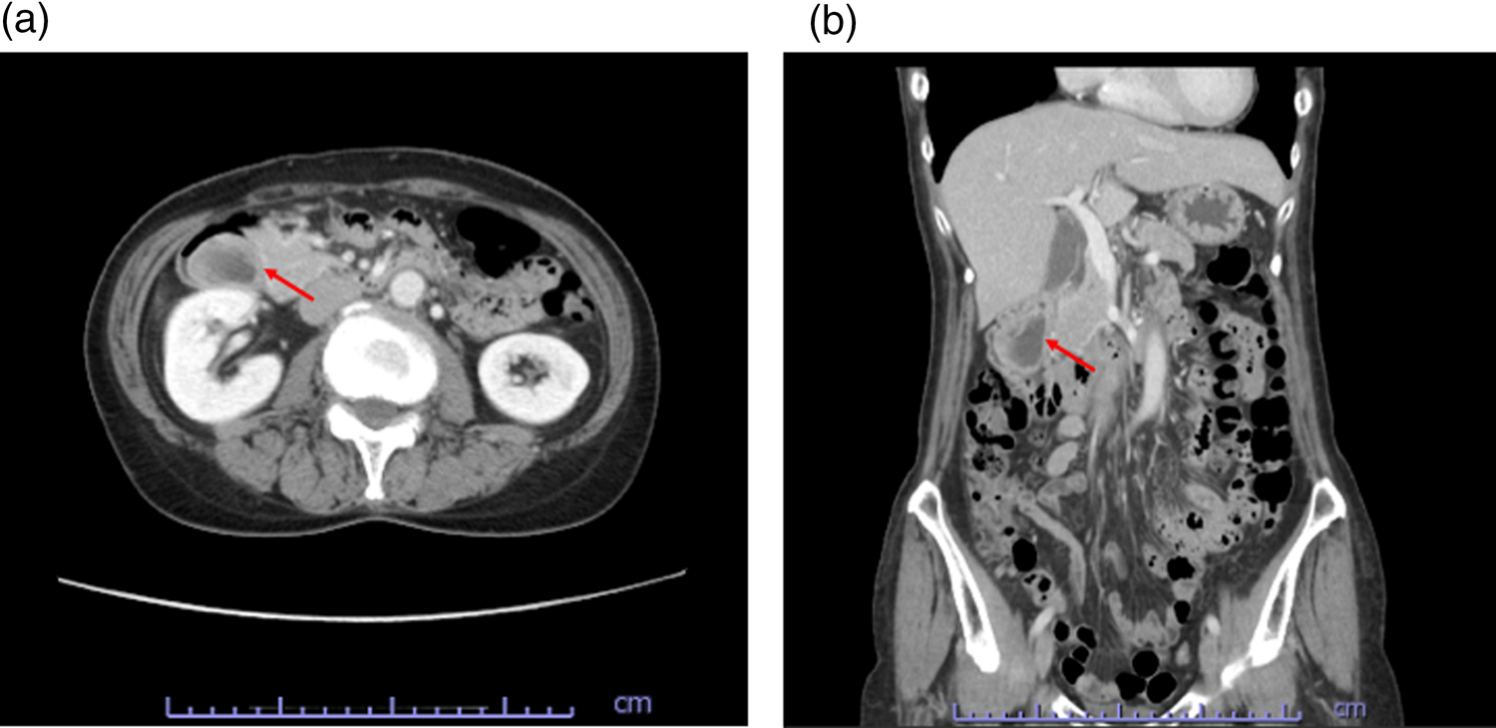
Abdominal computed tomography findings computed tomography demonstrated (red arrows) a 40‐mm pedunculated mass with internal nonuniformity in the descending part of the duodenum. (a) coronal image and (b) axial image

**FIGURE 3:**
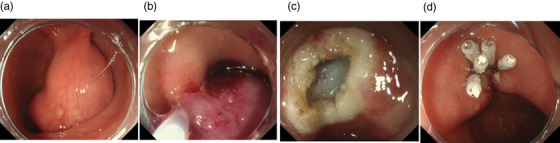
Endoscopic mucosal resection (EMR) images: (a) Duodenal submucosal tumor (SMT) on the anterior wall of the duodenal bulb and (b) EMR was performed to remove the duodenal SMT. Glycerol was injected locally to lift the basal mucosa and the lesion was resected using a snare. (c) Duodenal surface after resection of the tumor and (d) endoscopic clipping was successful

**FIGURE 4:**
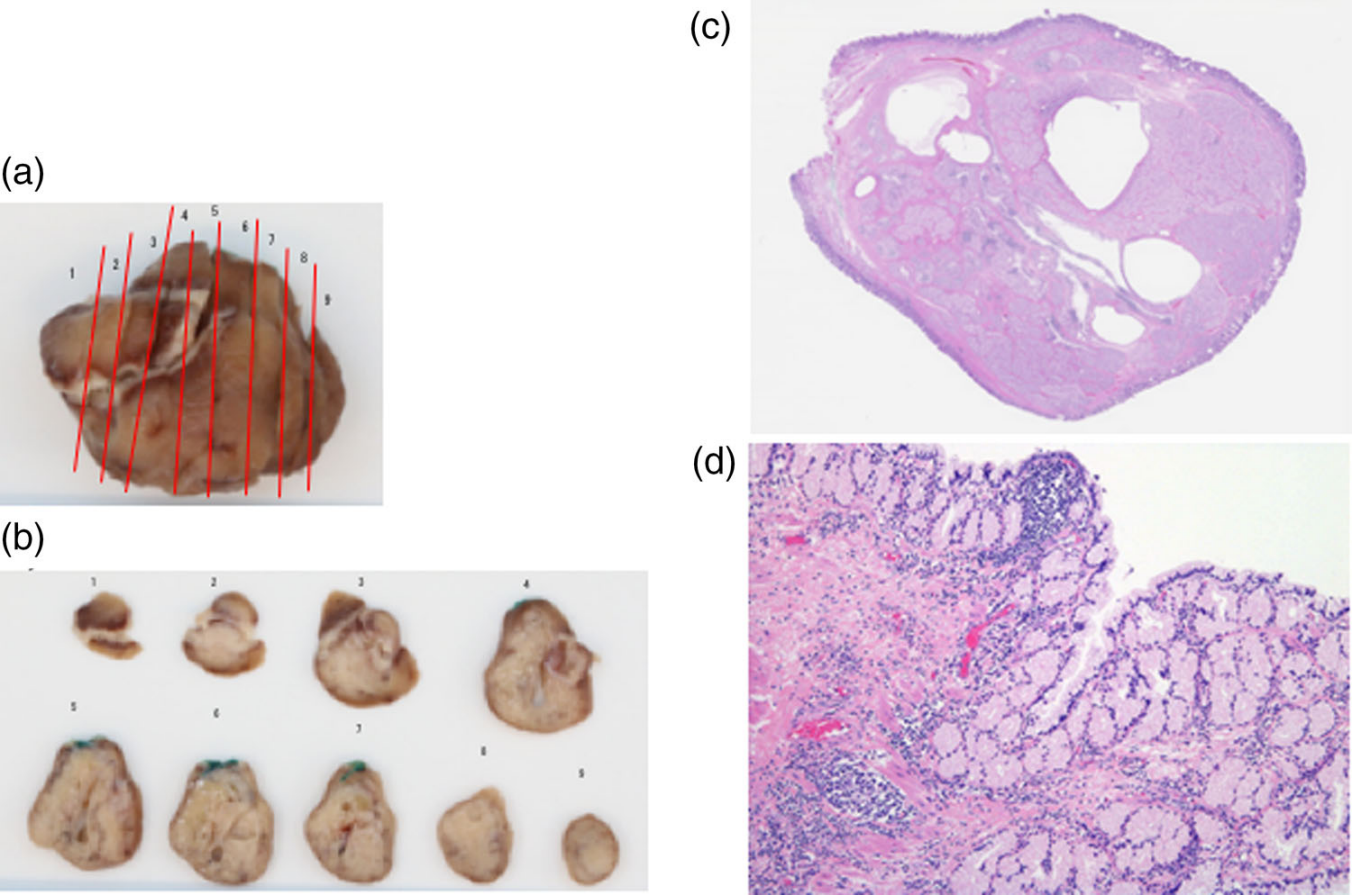
Pathological findings of the resected specimen (a) Specimen was 40 mm in diameter. The mucosal surface was smooth with glandular characteristics. (b) Macroscopic appearance of the cut surface of the resected specimen. (c) Histopathological examination of the resected tumor on a loupe view demonstrated a well‐defined pedunculated polypoid lesion with cystic structures. (d) Histopathological examination of the tumor on high‐power magnification revealed that there was a dense proliferation of Brunner's glands with no nuclear and structural atypia and irregular intercalation of smooth muscle bundles

The tumor diameter increased from 25 to 40 mm in 2 years (Figure 1a,b). Further, CT findings revealed a tumor in the duodenal bulb, 40 mm in diameter, with internal non‐uniform contrast enhancement (Figure 2a,b). We performed a thorough examination because of the increasing size of the tumor. EUS‐FNAB was performed using a 22‐gauge aspiration needle, which revealed a 40‐mm hypoechoic lesion, suspected to be a cyst with a solid hyperechoic component (Figure 1c). Histopathological examination revealed Brunner's glands and smooth muscle tissues in the preoperative biopsy specimen (Figure 1d). A final preoperative diagnosis of a duplication cyst was established based on the imaging findings, including those of EUS, and histopathological findings. The preoperative CT images showed abundant tumor blood flow, necessitating sufficient coagulation during resection. In addition, the duodenum is thin‐walled with a perforation risk, including delayed perforation.^4^ Thus, we chose EMR to ensure sufficient distance from the muscularis propria during EMR. EMR was performed because of the presence of clinical symptoms, such as intermittent abdominal pain, increase in tumor diameter, and risk of malignant transformation of the duplication cyst (Figure 3). However, histopathological examination of the resected tumor on a loupe view demonstrated a well‐defined pedunculated polypoid lesion with cystic components. The lesion was composed of a dense proliferation of Brunner's glands with lobular and cystic structure and irregular intercalation of smooth muscle bundles (Figure 4c,d). There was no nuclear or structural atypia in the Brunner's glands (Figure 4d). Therefore, we diagnosed the lesion as BGH.

The patient had epigastric pain since childhood. Her postoperative condition is good.

## DISCUSSION

BGH is an extremely rare disease that usually involves the following sites: duodenal bulb, 70%; second (descending) part, 26%; and third (inferior) part of the duodenum, 4%. Tumors sized ≥2 cm in diameter can present with symptoms of upper gastrointestinal bleeding or obstruction.^2^ Brunner's gland hyperplasia is described as multiple small polypoid or nodular lesions, in which a large number of Brunner's glands are separated by fibrous septa. BGH refers to a single mass consisting of Brunner's gland, fibrous septa, ducts, smooth muscle, adipose, and lymphoid tissue. According to the Armed Forces Institute of Pathology guidelines, a hyperplasia diagnosis is made when solitary or multiple lesions measuring <5 mm are present, whereas a hamartoma is diagnosed if the lesion size is ≥5 mm.

Largely isolated lesions that are symptomatic may be clinically indicative.^2^ In one case, pancreaticoduodenectomy was performed for suspected malignancy because of an increase in tumor size prior to surgery; however, postoperative histopathological analysis revealed BGH,^5^ which can be differentiated from leiomyoma, adenoma, lipoma, leiomyosarcoma, and carcinoid by performing EMR.^3^, ^6^, ^7^ Some studies have suggested that BGH may have malignant potential; however, recurrence of BGH has not been reported.^8^ Nevertheless, endoscopic resection may be an option in symptomatic patients or increasing tumor size.^3^ In this case, the patient had occasional abdominal pain since childhood, possibly because the tumor had a stalk and was movable. Although there was temporary upper abdominal pain because of increased pressure in the intestinal tract, the symptoms did not persist because the tumor was released, meaning there was no change in symptoms. Moreover, it was considered that a stalk was elongated and enlarged with the passage of food and peristaltic movement. Atypical neoplastic lesions with malignant potentials, such as neuroendocrine neoplasms, including duodenum gangliocytic paraganglioma, gastrointestinal stromal tumors, and sarcoma, were also identified.^6^ EUS‐FNAB was performed prior to endoscopic resection to make a differential diagnosis from malignant potential lesions that require invasive surgery, such as pancreaticoduodenal resection with lymph node dissection. The diagnostic ability and safety of EUS‐FNAB have been reported previously.^7^ We selected ER, a minimally invasive treatment, because of the tissue sampling.

Smooth muscle bundles are present among the dense proliferation of Brunner's glands in BGH. Similarly, a smooth muscle tissue was observed in the deeper layers of BGH in this case, which was the reason for the preoperative diagnosis of the duplication cyst. We believe that the irregular arrangement of blood vessels in the smooth muscle tissue was the cause of uneven contrast enhancement observed on CT. On histopathology, it was observed that the smooth muscle tissue and Brunner's gland were not uniformly distributed like mirror images; however, the smooth muscle tissue was admixed with a dense growth of Brunner's glands. Therefore, the final diagnosis was BGH rather than a duplication cyst.

BGH and duplication cysts are congenital diseases. Duplication cysts have similar presentation, with gastrointestinal symptoms, such as abdominal pain and vomiting,^9^ and histopathological findings of Brunner's glands and smooth muscles. Histologically, BGH is defined by the heterogeneous, hypoechoic appearance that contains small cystic areas with indistinct borders. Brunner's glands present as simple cystic, polycystic, or indistinct echogenic masses in the mucosa and submucosa, with hematomatous tissue depending on the degree of cystic glandular formation in the Brunner's glands. As BGH resembles demarcated cystic mass, it should be differentiated from a duplication cyst.^10^ Therefore, it is difficult to make a diagnosis based on clinical symptoms or EMR results alone. A previous report indicated that symptomatic and difficult‐to‐diagnose cases of BGH with malignant potential exist,^8^ and a minimally invasive treatment with EMR is thought to be part of the diagnostic process. Although dysplasia was not observed in this case, a long‐term EMR follow‐up examination is required. The number of patients undergoing extensive investigations and EMR has increased recently, and such duodenal tumors could be identified more frequently. Therefore, there is a need to focus on the presence of hamartomas when large submucosal tumors are identified.

In conclusion, we report a rare case of BGH that could not be diagnosed preoperatively, which could only be diagnosed after EMR via histopathological analysis of the resected tissue.

## CONFLICT OF INTEREST

The authors declare no conflict of interest. The author T.I. is an EIC of *DEN Open*.

## FUNDING INFORMATION

The authors received no funding from external sources for this manuscript.

## ETHICAL STATEMENT

This study was conducted in accordance with the ethical standards laid down in the 1964 Declaration of Helsinki and its later amendments.

## PATIENT CONSENT

We obtained written consent from the patient prior to publication of the case.

## References

[1] Botsford TW , Crowe P , Crocker DW . Tumors of the small intestine. A review of experience with 115 cases including a report of a rare case of malignant hemangio–endothelioma. Am J Surg 1962; 103: 358–65. 13871677 10.1016/0002-9610(62)90226-x

[2] Patel ND , Levy AD , Mehrotra AK , Sobin LH . Brunner's gland hyperplasia and hamartoma: Imaging features with clinicopathologic correlation. AJR Am J Roentgenol 2006; 187: 715–22. 16928936 10.2214/AJR.05.0564

[3] Kitagawa Y , Osumi H , Kawachi H *et al*. Giant duodenal Brunner's gland hamartoma successfully treated via endoscopic mucosal resection. Arab J Gastroenterol 2018; 19: 125–9. 30243896 10.1016/j.ajg.2018.08.004

[4] Nonaka S , Oda I , Tada K *et al*. Clinical outcome of endoscopic resection for nonampullary duodenal tumors. Endoscopy 2015; 47: 129–35. 25314330 10.1055/s-0034-1390774

[5] Stewart ZA , Hruban RH , Fishman EF , Wolfgang CL . Surgical management of giant Brunners's gland hamartoma: Case report and literature review. World J Surg Oncol 2009; 7: 68. 19725968 10.1186/1477-7819-7-68 PMC2749032

[6] Okubo Y . Gangliocytic paraganglioma: An overview and future perspective. World J Clin Oncol 2019; 10: 300–2. 31572665 10.5306/wjco.v10.i9.300 PMC6766464

[7] Itoi T , Tsuchiya T , Itokawa F , Sofuni A , Kurihara T , Tsuji S , Ikeuchi N . Histological diagnosis by EUS‐guided fine‐needle aspiration biopsy in pancreatic solid masses without on‐site cytopathologist: A single‐center experience. Dig Endosc 2011; 23: 34–8. 21535198 10.1111/j.1443-1661.2011.01142.x

[8] Brookes MJ , Manjunatha S , Allen CA , Cox M . Malignant potential in a Brunner's gland hamartoma. Postgrad Med J 2003; 79:416–7. 12897224 10.1136/pmj.79.933.416 PMC1742741

[9] Gross RE , Ladd WE . The surgery of infancy and childhood. Philadelphia and London: W.B. Saunders Company; 1953.

[10] Hizawa K , Iwai K , Esaki M *et al*. Endosonographic features of Brunner's gland hamartomas which were subsequently resected endoscopically. Endoscopy 2002; 34: 956–8. 12471538 10.1055/s-2002-35849

